# Real-world clinical and economic outcomes associated with supplemental oxygen therapy use among patients with fibrosing interstitial lung disease in the United States

**DOI:** 10.1186/s12890-025-03909-1

**Published:** 2025-11-14

**Authors:** Joseph Yang, Andrea Steffens, Lee Brekke, Amy Anderson, Gursimran Basra, Amy L. Olson, Phani Veeranki, Joao A. de Andrade

**Affiliations:** 1 Boehringer Ingelheim Pharmaceuticals, Inc, Ridgefield, CT USA; 2 Optum, Eden Prairie, MN USA; 3 Vanderbilt University Medical Center, Nashville, TN USA

**Keywords:** Idiopathic pulmonary fibrosis, Interstitial lung disease, Oxygen therapy, Economic burden

## Abstract

**Introduction:**

Patients with fibrosing interstitial lung disease (ILD) experience a decline in lung function with progressive symptoms, poor response to treatment, and reduced quality of life. While supplemental oxygen therapy is commonly prescribed in clinical practice for patients with fibrosing ILD, the long-term outcomes associated with oxygen therapy remain unclear. This study aimed to address this knowledge gap.

**Methods:**

This non-interventional study used the Optum^®^ Market Clarity database from 01 October 2015 to 30 June 2022. Patients aged ≥ 18 years with newly diagnosed fibrosing ILD (≥ 2 fibrosing ILD diagnoses on different service dates within 365 days) were included. Patients meeting initial selection criteria were assigned to cohorts based on oxygen therapy initiation. Patients who initiated oxygen therapy following the ILD diagnosis (oxygen therapy cohort) were propensity scores matched 1:1 to those who did not yet initiate oxygen therapy (no oxygen therapy cohort). The oxygen cohort’s index date was the first oxygen therapy date. For the no oxygen therapy cohort, it was assigned using the time between the fibrosing ILD diagnosis date and the index date of a matched oxygen therapy patient. Follow-up continued until health plan disenrollment, death, or end of study period (follow-up period).

**Results:**

A total of 24,680 patients who initiated oxygen therapy were successfully matched to those who did not. The mean age of the study cohort was 68.9 years and 50.9% were male. Mortality was significantly higher in the oxygen cohort vs. the no oxygen cohort (54.0% vs. 26.3%, *p* < 0.001). Similar trends were observed for hospitalizations and probable acute exacerbations. The oxygen cohort had significantly higher all-cause healthcare resource utilization (HCRU) across all categories and incurred greater total all-cause healthcare costs. Similar patterns were seen in fibrosing ILD-related HCRU and costs.

**Conclusions:**

Initiation of oxygen therapy in patients with fibrosing ILD is associated with significantly higher mortality, increased hospitalizations, and greater healthcare utilization and costs. However, these findings do not imply that oxygen therapy causes harm; rather, oxygen use likely reflects more advanced or rapidly progressing ILD. These results highlight the need for earlier and more effective interventions to delay disease progression and reduce the burden of care.

**Supplementary Information:**

The online version contains supplementary material available at 10.1186/s12890-025-03909-1.

## Introduction

Interstitial lung disease (ILD) comprises over 200 distinct lung disorders with various etiologies that are characterized by varying levels of inflammation and fibrosis of the interstitium of the lung [1]. Patients with ILD exhibit common respiratory-related symptoms, including dyspnea and persistent dry cough, which can lead to exertional limitation, hypoxemia, respiratory failure, and death [1, 2]. Among these, a subset of patients with fibrosing ILD develop a progressive phenotype marked by irreversible lung function decline, worsening symptoms, and reduced quality of life, despite standard therapy. Idiopathic pulmonary fibrosis (IPF) is the most common type of progressive ILD without a known cause [3]. Recent studies have underscored the importance of unresolved acute lung inflammation in driving fibrotic changes in the lung [3]. In addition, certain comorbidities such as gastroesophageal reflux disease (GERD) may further drive fibrogenesis through chronic microaspiration, impacting disease progression and prognosis [4]. Recent evidence highlights that patients with progressive fibrosing ILD, including non-IPF subtypes, may share similar clinical trajectories and outcomes, prompting a shift toward phenotype-based management strategies [5]. 

The major treatment goals in ILD are to slow the lung function decline and improve the symptoms, exercise capacity, and health-related quality of life [6]. Current guideline recommended pharmacologic treatment for ILD includes the antifibrotic agents nintedanib and pirfenidone [7, 8]. Both nintedanib and pirfenidone are indicated for the treatment of IPF, whereas nintedanib has additional indications as a treatment for progressive non-IPF ILD (i.e., progressive pulmonary fibrosis [PPF]) and systemic sclerosis-associated ILD (SSc-ILD). Moreover, immunosuppressants are often used to treat certain forms of PPF, such as those related to connective tissue diseases and hypersensitivity pneumonitis [9]. Despite these therapies, many patients continue to experience progressive clinical deterioration and reduced quality of life [10, 11]. 

Exertional and resting hypoxemia are common clinical events and are poor prognostic factors among patients with fibrosing ILD [12]. Hypoxemia is accompanied by increased dyspnea and reduced exercise capacity, which further decreases patients’ quality of life. In our recently published study, we found that close to 40% of patients with fibrosing ILD initiated oxygen therapy following their diagnosis [13]. Several clinical guidelines also recommend its use among ILD patients [14–16], however, evidence supporting its use is largely derived from studies on chronic obstructive pulmonary disease (COPD) and based on expert opinions. This highlights the need for studies on the efficacy and long-term effects of oxygen therapy in patients with fibrosing ILD to further refine these recommendations [17]. 

Long-term oxygen therapy has been suggested to offer benefits such as improved exercise capacity and enhanced health-related quality of life [18–20]. Despite these potential advantages, the evidence regarding its impact on dyspnea reduction and patient survival remains inconclusive, necessitating further research [21, 22]. Moreover, there are challenges associated with oxygen therapy that may counterbalance its long-term benefits, including logistical and financial barriers [23]. Therefore, a deeper understanding of the outcomes related to oxygen therapy is crucial. Building on our prior work that characterized oxygen therapy use in fibrosing ILD, this study leverages real-world data to evaluate the clinical and economic outcomes associated with its initiation [13]. By evaluating the clinical and economic outcomes associated with supplemental oxygen therapy use in patients with fibrosing ILD, we aim to fill these knowledge gaps.

## Methods

This study’s methodology builds upon our previously published manuscript that assessed the prevalence of oxygen therapy use among patients with fibrosing ILD [13]. To ensure methodological consistency, we applied similar patient selection criteria and data source as described in our previous work. This approach allows us to extend our previous findings by evaluating outcomes associated with oxygen therapy initiation [13]. 

### Data sources

This retrospective cohort study leveraged Optum’s de-identified Market Clarity Data (Optum^®^ Market Clarity), which includes integrated administrative claims and electronic health record (EHR) data. The database is statistically de-identified under the Health Insurance Portability and Accountability Act of 1996 (HIPAA) Privacy Rule’s Expert Determination method and is managed according to Optum^®^ customer data use agreements. The Optum^®^ Market Clarity dataset links EHR data with administrative claims data, including pharmacy claims, physician claims, facility claims (with clinical information), medications prescribed and administered, diagnoses, procedures, and information derived from clinical notes using natural language processing. In this study, only the subset of individuals with health plan enrollment data during the period from 01 October 2015 through 30 June 2022 (study period) were included.

### Study population

The study cohort of interest was patients with fibrosing ILD who initiated oxygen therapy after diagnosis. Adult patients (≥ 18 years of age) newly diagnosed with fibrosing ILD were identified from 01 October 2016 through 30 June 2022 (patient identification period). Newly diagnosed fibrosing ILD was defined as ≥ 2 fibrosing ILD diagnoses on different dates of service and within 365 days of each other, with the earliest date of a claim with ILD diagnosis set as the fibrosing ILD diagnosis date. To collect patient demographics and clinical characteristics, 12 months of continuous enrollment in a health plan prior to the fibrosing ILD diagnosis date (pre-ILD baseline period) was required for all patients. Patients with a fibrosing ILD diagnosis in the pre-ILD baseline period or with unknown gender or geographic region were excluded.

Patients meeting the initial selection criteria were stratified further based on the use of oxygen therapy after ILD diagnosis. Patients who initiated oxygen therapy and did not have evidence of oxygen therapy use during the pre-ILD baseline period (oxygen therapy cohort) were propensity scores matched to those who had not yet initiated oxygen therapy (no oxygen therapy cohort). The index date for the oxygen therapy cohort was defined as the first date of a claim for oxygen therapy. For the no oxygen therapy cohort, the index date was assigned to match the duration between the initial ILD diagnosis date and the index date of the matched oxygen therapy patient. The pre-index period was defined as the 12-month period prior to the index date, which may have included the fibrosing ILD diagnosis date. Follow-up for each patient continued until the earliest of the following events: disenrollment from the health plan, death, end of the study period, or initiation of oxygen therapy for a no oxygen therapy cohort patient.

### Study measures

#### Demographic and clinical characteristics

Patient demographic and clinical characteristics included age as of the index year, gender, race, insurance type, US Census region, and the Quan-Charlson comorbidity score, based on diagnosis codes on medical claims [24, 25]. General comorbid conditions were defined with Clinical Classifications Software managed by the Agency for Healthcare Research and Quality (AHRQ) [26]. Provider specialty, medication use (corticosteroids, biologics, calcineurin inhibitors, other immunosuppressants) were also assessed.

#### Outcomes

Clinical outcomes of interest included time to all-cause mortality, all-cause hospitalization, and probable acute exacerbations. A claims-based algorithm was used to identify probable acute exacerbation, which was defined as meeting all of the criteria: 1) ≥ 1 claim with procedure code for CT scan, 2) evidence of dyspnea within 30 days prior to the date of CT scan, 3) no claims with diagnosis for pulmonary infection, left heart failure, pulmonary embolism or other identifiable causes of acute lung injury within 30 days prior to the date of CT scan, and 4) evidence of high-dose corticosteroid therapy within 15-day window before or after the date of CT scan.

Economic outcomes included all-cause and fibrosing ILD-related health care resource utilization (HCRU) and direct health care costs, measured during the pre-ILD baseline, pre-index, and follow-up periods. HCRU included rate of ambulatory visits (physician office and hospital outpatient), emergency department (ER) visits, inpatient admissions, and pharmacy fills. Health care costs were calculated and presented in categories of pharmacy costs and medical costs (ambulatory [office visits, outpatient visits], ER costs, inpatient stay costs, other medical costs). Health care costs were adjusted to reflect inflation using the annual medical care component of the Consumer Price Index (CPI) [27]. Costs and HCRU were considered fibrosing ILD-related if the claim had a diagnosis in any position for fibrosing ILD, prescription claim for antifibrotic agents, or chest imaging tests (i.e., chest radiography, high-resolution computed tomography).

### Statistical analysis

#### Propensity score matching

To address potential confounding between study outcomes and oxygen therapy status, the oxygen therapy cohort was matched via propensity scores in a 1:1 ratio to the no oxygen therapy cohort. Propensity scores were estimated using the variables measured in the pre-ILD baseline period (fibrosing ILD diagnosis year, age, gender, race, ethnicity, insurance type, comorbidity, medication use, and healthcare resource utilization). For each oxygen therapy cohort patient, a patient from the no oxygen therapy cohort with the closest available propensity score, within a caliper of ± 0.01, was selected. Patients from the oxygen therapy cohort could serve as a no oxygen therapy control for another patient from the oxygen therapy cohort prior to their oxygen therapy initiation. In such instances, the follow-up for these patients concluded one day before they initiated oxygen therapy.

The quality of the match was evaluated by comparing the characteristics included in the propensity score model between cohorts using standardized mean differences (SMD). An SMD of less than 10% was considered an adequate balance between the cohorts. Statistical tests between cohorts included variance adjustments for clustering due to matching. Unmatched patients were excluded from the analysis.

#### Descriptive analyses

Numbers and percentages were provided for categorical variables; means and standard deviations (SD) were provided for continuous variables. Clinical measures were also reported for the pre-index period to capture any changes in patient status between the fibrosing ILD diagnosis date and the index date. HCRU and cost outcomes were weighted by the minimum follow-up time of each matched pair and reported as weighted per-patient-per-month (wPPPM). Kaplan-Meier analysis was used to estimate the censor-adjusted proportion of patients with all-cause mortality, probable acute exacerbation, and inpatient visits over time.

#### Multivariable analyses 

Multivariable analyses were performed for the key outcomes of interest, adjusting for pre-index characteristics. Differences between cohorts in all-cause and fibrosing ILD-related total and medical costs were assessed using generalized linear models with a gamma distribution and log link. Cox proportional hazards regression modeling was used to assess differences between cohorts in the hazard of all-cause mortality, probable acute exacerbation, and hospitalization. Three distinct hazard ratios (HR) and costs ratios were reported to account for significant interactions observed between cohort and inpatient setting at index date (i.e., hospitalized on the index date, hospitalized before and on the index date, not hospitalized on the index date).

## Results

### Pre-ILD baseline demographic and clinical characteristics

Among 93,578 patients meeting the initial cohort selection criteria, 24,686 (26.4%) initiated oxygen therapy after the fibrosing ILD diagnosis date (Supplementary Fig. 1). After matching, a total of 49,360 patients were included in the final study sample, of which 24,680 patients (50.0%) were included in the oxygen therapy cohort and 24,680 (50.0%) were included in the no oxygen therapy cohort.

The post-match baseline demographic and clinical characteristics were similar between the study cohorts (Table 1). The mean age at the initial ILD diagnosis was 68.9 years for both cohorts. In the matched oxygen therapy vs. no oxygen therapy cohorts, patients were predominantly male (51.2% vs. 50.6%; SMD = 1.01%), White (68.7% vs. 68.8%, SMD = 0.18%), from the Midwest (40.3% vs. 39.9%, SMD = 0.69%), and had Medicare insurance coverage (47.6% vs. 47.9%, SMD = 0.58%). The mean (SD) Charlson comorbidity score was 2.6 (2.4) and 2.5 (2.4), and COPD was the most commonly observed respiratory disease (37.0% vs. 37.6%, SMD = 1.22%) (Table 1).

**Table 1:** Post-match pre-ILD baseline patient demographic and clinical characteristics

Demographics	Oxygen therapy (N=24,680)	No oxygen therapy (N=24,680)	Standardized mean difference (%)
*Age (continuous)*	68.88	68.89	-0.09
Index age group, n (%)			
18-44	1,320 (5.35)	1,272 (5.15)	0.87
45-64	7,444 (30.16)	7,472 (30.28)	-0.25
65+	15,916 (64.49)	15,936 (64.57)	-0.17
*Gender, n (%)*			
Female	12,056 (48.85)	12,181 (49.36)	-1.01
Male	12,624 (51.15)	12,499 (50.64)	1.01
*Region, n (%)*			
Northeast	5,386 (21.82)	5,387 (21.83)	-0.01
Midwest	9,937 (40.26)	9,854 (39.93)	0.69
South	6,893 (27.93)	6,945 (28.14)	-0.47
West	2,464 (9.98)	2,494 (10.11)	-0.40
*Race, n (%)*			
White	16,955 (68.70)	16,976 (68.78)	-0.18
African-American	2,396 (9.71)	2,403 (9.74)	-0.10
Asian	350 (1.42)	342 (1.39)	0.28
Unknown	4,979 (20.17)	4,959 (20.09)	0.20
*Ethnicity, n (%)*			
Hispanic	980 (3.97)	1,009 (4.09)	-0.60
Not Hispanic	17,124 (69.38)	17,091 (69.25)	0.29
Unknown	6,576 (26.65)	6,580 (26.66)	-0.04
*Insurance type, n (%)*			
Commercial only	7,595 (30.77)	7,563 (30.64)	0.28
Medicare only	11,741 (47.57)	11,812 (47.86)	-0.58
Medicaid only	2,393 (9.70)	2,328 (9.43)	0.90
Multiple known types	2,248 (9.11)	2,260 (9.16)	-0.17
Unknown type	703 (2.85)	717 (2.91)	-0.34
*Fibrosing ILD year* ^*1*^ *, n (%)*			
2016	1,903 (7.71)	2,226 (9.02)	-4.73
2017	5,483 (22.22)	6,242 (25.29)	-7.23
2018	4,726 (19.15)	5,073 (20.56)	-3.53
2019	4,356 (17.65)	4,394 (17.80)	-0.40
2020	3,518 (14.25)	3,268 (13.24)	2.94
2021	3,677 (14.90)	2,938 (11.90)	8.80
2022	1,017 (4.12)	539 (2.18)	11.10
*Index year, n (%)*			
2016	500 (2.03)	832 (3.37)	-8.31
2017	3,382 (13.70)	4,064 (16.47)	-7.73
2018	4,093 (16.58)	4,456 (18.06)	-3.89
2019	4,577 (18.55)	4,683 (19.97)	-1.10
2020	4,365 (17.69)	4,335 (17.56)	0.32
2021	5,348 (21.67)	4,495 (18.21)	8.66
2022	2,415 (9.79)	1,815 (7.35)	8.69
Charlson comorbidity score^2^ (continuous), mean (SD)	2.58 (2.41)	2.53 (2.36)	2.33
*Charlson comorbidity score (categorical), n (%)*			
0	5,154 (20.88)	5,004 (20.28)	1.50
1-2	8,877 (35.97)	9,290 (37.64)	-3.47
3-4	5,885 (23.85)	5,907 (23.93)	-0.21
5+	4,764 (19.30)	4,479 (18.15)	2.96
*Underlying ILD type during the study period (mutually exclusive), n (%)*			
Autoimmune ILD	3,205 (12.99)	3,581 (14.51)	-4.43
Hypersensitivity Pneumonitis	6,492 (26.30)	6,099 (24.71)	3.65
Sarcoidosis	519 (2.10)	502 (2.03)	0.48
Multiple	1,333 (5.40)	1,586 (6.43)	-4.35
Unclassified IIP	13,131 (53.21)	12,912 (52.32)	1.78
IPF	2414 (9.78)	2,435 (9.87)	-0.29
*Medications, n (%)*			
Antifibrotics	22 (0.09)	24 (0.10)	-0.27
Corticosteroids	9,181 (37.20)	9,500 (38.49)	-2.67
Biologics	714 (2.89)	741 (3.00)	-0.65
Calcineurin inhibitors	237 (0.96)	223 (0.90)	0.59
Other immunosuppressants	824 (3.34)	928 (3.76)	-2.28
*Respiratory diseases, n (%)*			
Pulmonary hypertension	1,928 (7.81)	1,863 (7.55)	0.99
Acute respiratory failure	2,781 (11.27)	2,346 (9.51)	5.78
Asthma	3,967 (16.07)	4,106 (16.64)	-1.52
COPD	9,140 (37.03)	9,286 (37.63)	-1.22
Pneumonia	6,157 (24.95)	5,892 (23.87)	2.50
Lung transplantation	62 (0.25)	69 (0.28)	-0.55
Lung cancer	1,071 (4.34)	1,071 (4.34)	0.00
Cystic fibrosis	48 (0.19)	53 (0.21)	-0.45
Respiratory tract infection	8,033 (32.55)	8,222 (33.31)	-1.63
Upper respiratory tract infection	5,247 (21.26)	5,420 (21.96)	-1.70
Lower respiratory tract infection	4,516 (18.30)	4,662 (18.89)	-1.52
*Other clinical characteristics, n (%)*			
Gastroesophageal reflux disease (GERD)	8,221 (33.31)	8,193 (33.20)	0.24
Heart failure	5,872 (23.79)	5,601 (22.69)	2.60
Obstructive sleep apnea	4,452 (18.04)	4,540 (18.40)	-0.92
Smoking status, n (%)	10,294 (41.71)	10,152 (41.13)	1.17

Two-sample t-test was used for continuous measures

Z-test using robust standard errors in an OLS regression was used for continuous measures

Pearson chi-square test was used for binary measures

Rao-Scott test was used for binary measures

^1^The identification period used only partial years in 2016 (October – December) and 2022 (January – July)

^2^Ref. [25]

### Clinical and economic outcomes

#### Clinical outcomes

**Fig. 1:**
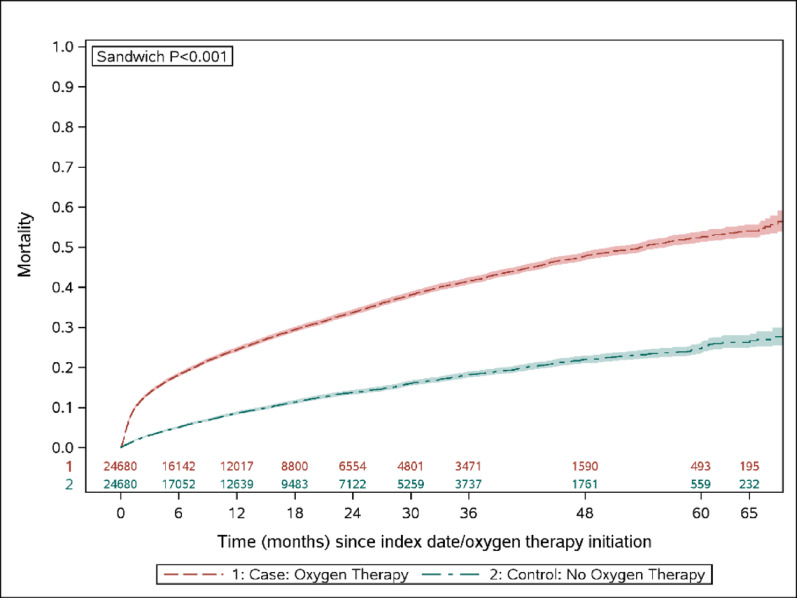
Time-to-all-cause mortality from the index date during follow-up

The mean (SD) length of follow-up among the oxygen therapy vs. no oxygen therapy was 16.6 (16.2) and 17.7 (16.4) months, respectively. The mean (SD) duration between the initial fibrosing ILD diagnosis and the index date for both cohorts was 8.5 (12.6) months. At 65 months, a significantly higher percentage of the oxygen therapy cohort had died compared to the no oxygen therapy cohort (Kaplan-Meier adjusted percentages of 54.0% vs. 26.3%, *p* < 0.001; Fig. 1). Similarly, a significantly higher percentage of the oxygen therapy cohort experienced hospitalization and probable acute exacerbation during the follow-up period (Supplementary Figs. 2 and 3).

**Fig. 2:**
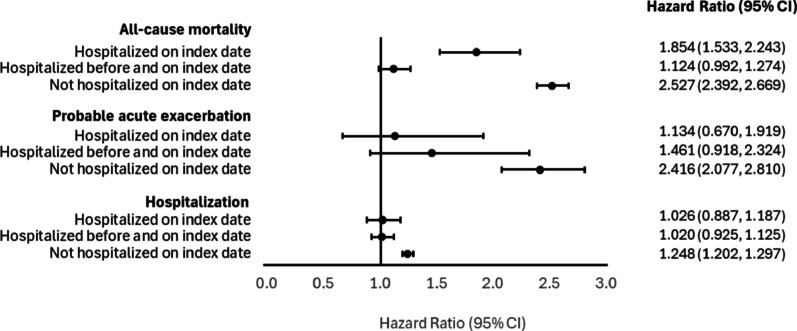
Adjusted hazard ratios ofall-cause mortality, probable acute exacerbation, and hospitalization, oxygen therapy vs. nooxygen therapy

We observed a significant difference in all-cause mortality risk between the cohorts using the Cox proportional hazards regression model, adjusting for the clinical characteristics measured during the pre-index period. Among patients who were not hospitalized on the index date, the oxygen therapy cohort had 2.5-fold higher risk of all-cause mortality vs. no oxygen therapy (HR = 2.52; 95% confidence interval [CI] = 2.39–2.67; Fig. 2). Similar trends were observed for hospitalization (HR = 1.24; 95% CI = 1.20–1.30) and probable acute exacerbation (HR = 2.41; 95% CI = 2.08–2.81). Among patients who were hospitalized on the index date, the oxygen therapy cohort had 85% higher risk of all-cause mortality vs. no oxygen therapy (HR = 1.85; 95% CI = 1.53–2.24; Fig. 2), whereas the adjusted HRs for probable acute exacerbation and hospitalizations were not statistically significant. Lastly, the adjusted HRs for all-cause mortality, probable acute exacerbation, and hospitalization were not statistically significant among patients who were hospitalized before and continued till the index date.

### Economic outcomes

During the follow-up period, the oxygen therapy cohort had significantly higher all-cause and fibrosing ILD-related HCRU compared to the no oxygen therapy cohort across all categories. The oxygen therapy cohort had higher wPPPM ambulatory visits (2.64 [2.81] vs. 2.30 [2.51], *p* < 0.001), emergency room visits (0.24 [0.52] vs. 0.17 [0.46], *p* < 0.001), and inpatient stays (0.09 [0.21] vs. 0.05 [0.16], *p* < 0.001) (Table 2).

**Table 2:** All-cause and fibrosing ILD-related health care resource utilization during the follow-up period

	Oxygen therapy (*N*=24,680)	No oxygen therapy(*N*=24,680)	*p*-value
*All-cause HCRU counts, mean (SD), wPPPM*			
Ambulatory visits	2.64 (2.81)	2.30 (2.51)	<0.001
Office visits	1.47 (1.67)	1.38 (1.52)	<0.001
Outpatient visits	1.20 (2.01)	0.93 (1.77)	<0.001
Emergency room visits	0.24 (0.52)	0.17 (0.46)	<0.001
Inpatient stays	0.09 (0.21)	0.05 (0.16)	<0.001
Length of stay, days	1.24 (4.35)	0.54 (3.46)	<0.001
Pharmacy fills	4.21 (4.10)	3.57 (3.85)	<0.001
*Fibrosing ILD-related HCRU counts, mean (SD), wPPPM*			
Ambulatory visits	0.34 (0.57)	0.21 (0.40)	<0.001
Office visits	0.13 (0.27)	0.09 (0.20)	<0.001
Outpatient visits	0.21 (0.44)	0.13 (0.32)	<0.001
Emergency room visits	0.08 (0.21)	0.05 (0.19)	<0.001
Inpatient stays	0.06 (0.18)	0.02 (0.11)	<0.001
Length of stay, days	0.98 (3.78)	0.36 (3.27)	<0.001
Pharmacy fills	0.02 (0.11)	0.01 (0.07)	<0.001

*HCRU* health care resource utilization, *wPPPM* weighted per-patient-per-month

Fibrosing-ILD-related is defined as any diagnosis of fibrosis or a claim for chest imaging or a claim for antifibrotic medication

**Fig. 3:**
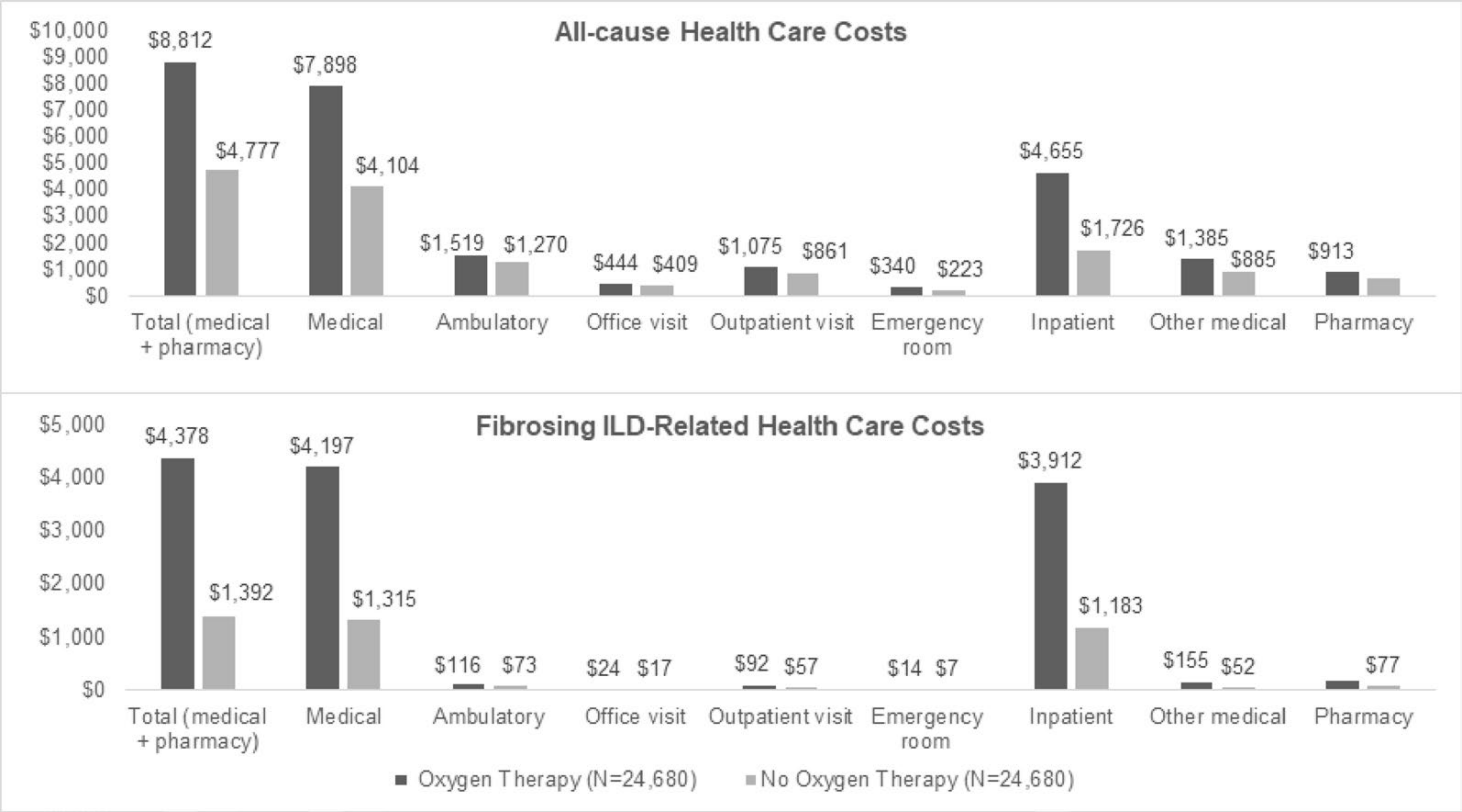
Follow-up period weighted all-cause andfibrosing ILD-related total costs

The mean (SD) wPPPM all-cause total costs were significantly higher in the oxygen therapy cohort vs. no oxygen therapy cohort ($8,812 [$27,142] vs. $4,777 [$13,571], *p* < 0.001; Fig. 3). The proportions of total costs attributable to medical costs were 89.6% and 85.9% in the oxygen therapy and no oxygen therapy cohorts, respectively. Inpatient costs were the major contributor to medical costs in both cohorts, accounting for 58.9% and 42.0% in oxygen therapy and no oxygen therapy cohorts, respectively. Similar trends were observed for fibrosing ILD-related costs.

**Fig. 4:**
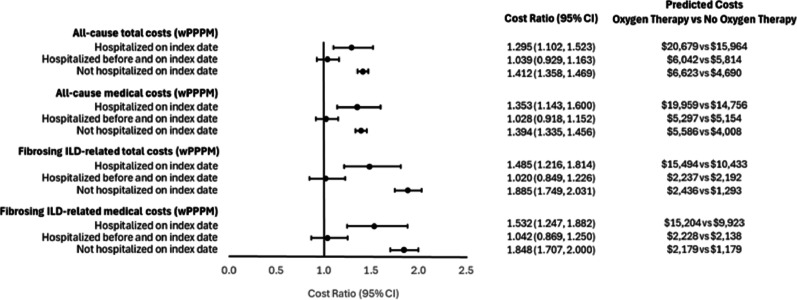
Multivariable Generalized Linear Regression Model: Adjusted cost ratios and predicted costs for all-cause and fibrosing ILD-related total and medical costs, oxygen therapy vs no oxygen therapy

We observed significant differences in cost ratios between the cohorts after adjusting for the pre-index characteristics. Among patients who were not hospitalized on the index date, the oxygen therapy cohort had 41% higher all-cause total costs (adjusted cost ratio [aCR] = 1.41; 95% CI = 1.36–1.47) and 39% higher all-cause medical costs (aCR = 1.39; 95% CI = 1.34–1.46) compared to the no oxygen therapy cohort (Fig. 4). Among patients who were hospitalized on the index date, the oxygen therapy cohort had 30% higher all-cause total costs (aCR = 1.30; 95% CI = 1.10–1.52) and 35% higher all-cause medical costs (aCR = 1.35; 95% CI = 1.14–1.60). Similar trends were observed for the fibrosing ILD-related total and medical costs (Fig. 4).

## Discussion

This study evaluated the long-term clinical and economic outcomes among patients with fibrosing ILD who initiated oxygen therapy compared to those who did not initiate oxygen therapy in the real-world setting. Our findings demonstrated that the oxygen therapy cohort had significantly higher rates of all-cause mortality, hospitalizations, and probable acute exacerbations during the follow-up period compared to the no oxygen therapy cohort. Furthermore, the oxygen therapy cohort had higher HCRU and incurred higher total healthcare costs compared to no oxygen therapy cohort. The study findings remained consistent after multivariate analyses with adjustment for pre-index characteristics.

To our knowledge, this is the first real-world study to comprehensively evaluate both clinical and economic outcomes associated with oxygen therapy in patients with fibrosing ILD. Our findings are aligned with prior observational studies that suggested an association between oxygen therapy and disease progression [28–30]as well as an increased risk of death or lung transplant [30]. From a clinical standpoint, it is possible that the higher rates of adverse clinical outcomes among oxygen users are merely a reflection of disease progression and severity. In fact, previous studies have employed oxygen therapy use as a proxy for ILD disease progression in studies using administrative claims data [31, 32] .

Interestingly, when stratifying by hospitalization status at or before the index date, the association between oxygen therapy and adverse outcomes was attenuated, with hazard ratios for most outcomes no longer statistically significant. This suggests that hospitalization itself may be a strong indicator of disease severity, potentially overshadowing the prognostic value of oxygen therapy for clinical outcomes. This implies once patients reach a certain level of disease severity, as reflected by recent or ongoing hospitalizations, the additional risk being associated with oxygen therapy becomes less apparent. This highlights the importance of considering hospitalization as a co-occurring marker of disease progression when interpreting the impact of oxygen therapy.

This study adds novel insights relevant for policy and population health decision makers by quantifying the incremental healthcare burden associated with oxygen therapy in this population, using a large, nationally representative real-world dataset. Our study findings provide additional evidence and reinforce that patients with progressive fibrosing ILD have higher HCRU and incur greater health care costs compared to those without progression [31-34] .

Moreover, initiation of oxygen therapy is an important landmark in a patient’s clinical trajectory, and previous research indicates that supplemental oxygen therapy is correlated with decreased quality of life across various domains, such as emotional well-being, independence, and social participation [35-39] . The DISCOVERY study revealed complex patterns regarding outcomes among patients with ILD who initiated oxygen therapy. The initial study results demonstrated that patients with ILD experienced increased acute exacerbations and hospitalizations following long-term oxygen therapy initiation over a 12-month period. However, sensitivity analyses of ILD patients with adequate 12-month follow-up data demonstrated the opposite effect—showing reduced acute exacerbations and all-cause hospitalizations [40] . These divergent findings underscore the complexity of oxygen therapy’s impact in ILD, suggesting that outcomes may vary significantly based on care quality, follow-up duration, and patient selection criteria. Collectively, these results support the clinical relevance of oxygen therapy as a marker of disease severity and healthcare burden, even as its direct therapeutic effects remain complex and context dependent. Therefore, delaying the need for oxygen therapy may represent a clinically meaningful goal. Therapies aimed at delaying or reducing the lung function decline and ILD progression, hence the need for oxygen therapy use in patients with ILD have the potential to improve clinical and economic outcomes in this population. Continued research and investment in such therapeutic options are warranted, and future clinical trials ought to consider using it as an outcome measure.

### Limitations

This study has several limitations that need to be considered when interpreting the results. While we utilized robust matching procedures to balance the study cohorts, this study lacked access to clinical measures of disease severity, such as forced vital capacity (FVC), or radiological assessments which are typically available only through registries or prospective studies. As a result, we were unable to directly adjust for lung function decline. Future research using registry data or other sources that capture pulmonary function metrics will be important to validate and extend these findings. Additionally, identification of newly diagnosed fibrosing ILD patients relied on a claims-based algorithm, and the presence of a diagnosis code does not necessarily indicate presence of disease. To mitigate misidentification of ILD patients, a confirmatory diagnosis was required, and patients without ILD diagnosis in the 12-month pre-ILD baseline period were considered newly diagnosed. We also recognize that coding errors may result in inaccurate or incomplete data, leading to potential misclassification of variables of interest that could have biased our findings. Lastly, our study findings cannot be generalized to uninsured populations and patients with health plans not represented in the database.

## Conclusion

This study provides insights into the real-world clinical and economic burden experienced by patients with fibrosing ILD who initiate oxygen therapy. Patients with fibrosing ILD who initiated oxygen therapy are associated with a higher incremental economic burden, primarily driven by increased hospitalization costs, and experienced increased rates of adverse clinical outcomes compared to patients who did not initiate oxygen therapy. Importantly, this study does not suggest that oxygen therapy itself causes harm; rather, oxygen use likely reflects more advanced disease and may serve as a surrogate for poor prognosis. Future management strategies and novel therapies that reduce ILD progression and hence the need for oxygen therapy could potentially alleviate clinical and economic burden in this population additionally.

## Supplementary Information

Below is the link to the electronic supplementary material.

Supplementary file1 (DOCX 150 KB)

## Data Availability

The data contained in the database used for the study contains proprietary elements owned by Optum and, therefore, cannot be broadly disclosed or made publicly available at this time. The disclosure of these data to third parties assumes certain data security and privacy protocols are in place and that the third party has executed a standard license agreement which includes restrictive covenants governing the use of the data. For inquiries regarding access to the raw data analyzed in this study, please contact the corresponding author, Joseph Yang, at joseph.yang@boehringer-ingelheim.com. Please see https://business.optum.com/en/data-analytics/life-sciences.html for more information about licensing these data from Optum.

## References

[1] Travis WD, Costabel U, Hansell DM, et al. An official American thoracic society/european respiratory society statement: update of the international multidisciplinary classification of the idiopathic interstitial pneumonias. Am J Respir Crit Care Med. 2013;15(6):733–48. 10.1164/rccm.201308-1483ST. 10.1164/rccm.201308-1483ST PMC5803655 24032382

[2] Wijsenbeek MS, Holland AE, Swigris JJ, Renzoni EA. Comprehensive supportive care for patients with fibrosing interstitial lung disease. Am J Respir Crit Care Med. 2019;15(2):152–9. 10.1164/rccm.201903-0614PP. 10.1164/rccm.201903-0614PP 31051080

[3] Savin IA, Zenkova MA, Sen’kova AV. Pulmonary fibrosis as a result of acute lung inflammation: molecular mechanisms, relevant in vivo models, prognostic and therapeutic approaches. Int J Mol Sci. 2022;2 3(23). 10.3390/ijms232314959 36499287 10.3390/ijms232314959 PMC9735580

[4] Ruaro B, Pozzan R, Confalonieri P, et al. Gastroesophageal reflux disease in idiopathic pulmonary fibrosis: viewer or actor? To treat or not to treat? Pharmaceuticals (Basel). 2022; 15(8). 10.3390/ph15081033 36015181 10.3390/ph15081033 PMC9412643

[5] Wong AW, Ryerson CJ, Guler SA. Progression of fibrosing interstitial lung disease. Respir Res. 2020;29(1):32. 10.1186/s12931-020-1296-3. 10.1186/s12931-020-1296-3 PMC6988233 31996266

[6] Raghu G, Richeldi L. Current approaches to the management of idiopathic pulmonary fibrosis. Respir Med. 2017;129:24–30. 10.1016/j.rmed.2017.05.017. 28732832 10.1016/j.rmed.2017.05.017

[7] Raghu G, Rochwerg B, Zhang Y, et al. An official ATS/ERS/JRS/ALAT clinical practice guideline: treatment of idiopathic pulmonary fibrosis. An update of the 2011 clinical practice guideline. Am J Respir Crit Care Med. Jul 2015;15(2):e3–19. 10.1164/rccm.201506-1063ST. 10.1164/rccm.201506-1063ST 26177183

[8] Raghu G, Remy-Jardin M, Richeldi L, et al. Idiopathic pulmonary fibrosis (an Update) and progressive pulmonary fibrosis in adults: an official ATS/ERS/JRS/ALAT clinical practice guideline. Am J Respir Crit Care Med. 2022;1(9):e18–47. 10.1164/rccm.202202-0399ST. 10.1164/rccm.202202-0399ST PMC9851481 35486072

[9] Copeland CR, Lancaster LH. Management of progressive fibrosing interstitial lung diseases (PF-ILD). Front Med (Lausanne). 2021;8:743977. 10.3389/fmed.2021.743977. 34722582 10.3389/fmed.2021.743977 PMC8548364

[10] Lindell KO, Liang Z, Hoffman LA, et al. Palliative care and location of death in decedents with idiopathic pulmonary fibrosis. Chest. 2015;147(2):423–9. 10.1378/chest.14-1127. 25187973 10.1378/chest.14-1127 PMC4314817

[11] Liang Z, Hoffman LA, Nouraie M, et al. Referral to palliative care infrequent in patients with idiopathic pulmonary fibrosis admitted to an intensive care unit. J Palliat Med. 2017;20(2):134–40. 10.1089/jpm.2016.0258. 27754815 10.1089/jpm.2016.0258

[12] Khor YH, Gutman L, Abu Hussein N, et al. Incidence and prognostic significance of hypoxemia in fibrotic interstitial lung disease: an international cohort study. Chest. 2021;160(3):994–1005. 10.1016/j.chest.2021.04.037. 33905679 10.1016/j.chest.2021.04.037

[13] Yang J, Steffens A, Olson AL, et al. Supplemental oxygen therapy use among patients with fibrosing interstitial lung disease in the united States. Respir Res. 2025;28(1):80. 10.1186/s12931-025-03139-3. 10.1186/s12931-025-03139-3 PMC11871663 40022082

[14] Raghu G, Collard HR, Egan JJ, et al. An official ATS/ERS/JRS/ALAT statement: idiopathic pulmonary fibrosis: evidence-based guidelines for diagnosis and management. Am J Respir Crit Care Med. 2011;15(6):788–824. 10.1164/rccm.2009-040GL. 10.1164/rccm.2009-040GL PMC5450933 21471066

[15] Bradley B, Branley HM, Egan JJ, et al. Interstitial lung disease guideline: the British Thoracic Society in Collaboration with the Thoracic Society of Australia and New Zealand and the Irish Thoracic Society. Thorax. 2008;63(Suppl 5):v1–58. 10.1136/thx.2008.101691. 18757459 10.1136/thx.2008.101691

[16] Hardinge M, Annandale J, Bourne S, et al. British Thoracic Society Guidelines for home oxygen use in adults. Thorax. 2015;70(Suppl 1):i1–43. 10.1136/thoraxjnl-2015-206865. 25870317 10.1136/thoraxjnl-2015-206865

[17] Lin LY, Wu YC, Wu JS, Tai HY, Huang TW, Cheng WH. Oxygen therapy for exercise capacity in fibrotic interstitial lung disease: a systematic review and meta-analysis of randomised controlled trials. Respir Med. 2024;227:107657. 10.1016/j.rmed.2024.107657. 38718907 10.1016/j.rmed.2024.107657

[18] Visca D, Mori L, Tsipouri V, et al. Effect of ambulatory oxygen on quality of life for patients with fibrotic lung disease (AmbOx): a prospective, open-label, mixed-method, crossover randomised controlled trial. Lancet Respir Med. 2018;6(10):759–70. 10.1016/s2213-2600(18)30289-3. 10.1016/S2213-2600(18)30289-3 30170904

[19] Tonga KO, Oliver BG. Effectiveness of pulmonary rehabilitation for chronic obstructive pulmonary disease therapy: focusing on traditional medical practices. J Clin Med. 2023;12(14):4815 . 10.3390/jcm12144815 10.3390/jcm12144815 PMC10381859 37510930

[20] Janssens JP, Rochat T, Frey JG, Dousse N, Pichard C, Tschopp JM. Health-related quality of life in patients under long-term oxygen therapy: a home-based descriptive study. Respir Med. 1997;91(10):592–602. 10.1016/s0954-6111(97)90005-6. 9488892 10.1016/s0954-6111(97)90005-6

[21] Bell EC, Cox NS, Goh N, et al. Oxygen therapy for interstitial lung disease: a systematic review. Eur Respir Rev. 2017;26(143):160080 . 10.1183/16000617.0080-2016 10.1183/16000617.0080-2016 PMC9489021 28223395

[22] Sharp C, Adamali H, Millar AB. Ambulatory and short-burst oxygen for interstitial lung disease. Cochrane Database Syst Rev. 2016;6(7):CD011716. 10.1002/14651858.CD011716.pub2 10.1002/14651858.CD011716.pub2 PMC6457989 27383922

[23] Clark KP, Degenholtz HB, Lindell KO, Kass DJ. Supplemental oxygen therapy in interstitial lung disease: a narrative review. Ann Am Thorac Soc. 2023;20(11):1541–9. 10.1513/AnnalsATS.202304-391CME. 37590496 10.1513/AnnalsATS.202304-391CME

[24] Bayliss EA, Ellis JL, Shoup JA, Zeng C, McQuillan DB, Steiner JF. Association of patient-centered outcomes with patient-reported and ICD-9-based morbidity measures. Ann Fam Med. 2012;10(2):126–33. 10.1370/afm.1364. 22412004 10.1370/afm.1364 PMC3315135

[25] Quan H, Li B, Couris CM, et al. Updating and validating the Charlson Comorbidity Index and score for risk adjustment in hospital discharge abstracts using data from 6 countries. Am J Epidemiol. 2011;15(6):676–82. 10.1093/aje/kwq433. 10.1093/aje/kwq433 21330339

[26] Agency for Healthcare Research and Quality. Clinical classification software (CCS) for ICD-10-CM. https://www.hcup-us.ahrq.gov/toolssoftware/ccs10/ccs10.jsp. Accessed February 22, 2023.

[27] US Department of Labor BoLS. Consumer Price Index. Medical Care. Series ID: CUUR0000SAM. Accessed September 17, 2023. http://data.bls.gov/cgi-bin/surveymost?cu

[28] Hook JL, Arcasoy SM, Zemmel D, Bartels MN, Kawut SM, Lederer DJ. Titrated oxygen requirement and prognostication in idiopathic pulmonary fibrosis. Eur Respir J. 2012;39(2):359–65. 10.1183/09031936.00108111. 21885386 10.1183/09031936.00108111 PMC3236811

[29] Ryerson CJ, Camp PG, Eves ND, et al. High oxygen delivery to preserve exercise capacity in patients with idiopathic pulmonary fibrosis treated with nintedanib. Methodology of the HOPE-IPF study. Ann Am Thorac Soc. 2016;13(9):1640–7. 10.1513/AnnalsATS.201604-267OC 27348402 10.1513/AnnalsATS.201604-267OC

[30] Snyder L, Neely ML, Hellkamp AS, et al. Predictors of death or lung transplant after a diagnosis of idiopathic pulmonary fibrosis: insights from the IPF-PRO registry. Respir Res. 2019;30(1):105. 10.1186/s12931-019-1043-9 10.1186/s12931-019-1043-9 PMC6542049 31142314

[31] Singer D, Bengtson LGS, Conoscenti CS, et al. Burden of illness in progressive fibrosing interstitial lung disease. J Manag Care Spec Pharm. Aug 2022;28(8):871-880. 10.18553/jmcp.2022.28.8.871 10.18553/jmcp.2022.28.8.871 PMC10373037 35876293

[32] Nili M, Steffens A, Anderson A, et al. Health care costs and utilization of progressive fibrosing lung disease by underlying interstitial lung disease type. J Manag Care Spec Pharm. Feb 3 2024;30(2):163-174. 10.18553/jmcp.2024.30.2.163 10.18553/jmcp.2024.30.2.163 PMC10839459 38308627

[33] Collard HR, Chen SY, Yeh WS, et al. Health care utilization and costs of idiopathic pulmonary fibrosis in U.S. Medicare beneficiaries aged 65 years and older. Ann Am Thorac Soc. Jul 2015;12(7):981-7. 10.1513/AnnalsATS.201412-553OC 10.1513/AnnalsATS.201412-553OC 25923447

[34] Olson AL, Maher TM, Acciai V, et al. Healthcare Resources Utilization and Costs of Patients with Non-IPF Progressive Fibrosing Interstitial Lung Disease Based on Insurance Claims in the USA. Adv Ther. Jul 2020;37(7):3292-3298. 10.1007/s12325-020-01380-4 10.1007/s12325-020-01380-4 PMC7467408 32451950

[35] Aronson K, Jacobs SS, Repola D, Swigris JJ. Is it time to include oxygen needs as an endpoint in clinical trials in patients with fibrosing interstitial lung disease? If so, how? BMJ Open Respir Res. Jul 2023;10(1):e001546 10.1136/bmjresp-2022-001546 37419519 10.1136/bmjresp-2022-001546 PMC10347448

[36] Lindell KO, Collins EG, Catanzarite L, et al. Equipment, access and worry about running short of oxygen: Key concerns in the ATS patient supplemental oxygen survey. Heart Lung. May-Jun 2019;48(3):245-249. 10.1016/j.hrtlng.2018.12.006 30598231 10.1016/j.hrtlng.2018.12.006

[37] Earnest MA. Explaining adherence to supplemental oxygen therapy: the patient's perspective. J Gen Intern Med. Oct 2002;17(10):749-55. 10.1046/j.1525-1497.2002.20218.x 12390550 10.1046/j.1525-1497.2002.20218.x PMC1495112

[38] Swigris JJ, Wilson H, Esser D, et al. Psychometric properties of the St George's Respiratory Questionnaire in patients with idiopathic pulmonary fibrosis: insights from the INPULSIS trials. BMJ Open Respir Res. 2018;5(1):e000278. 10.1136/bmjresp-2018-000278 10.1136/bmjresp-2018-000278 PMC5976110 29862029

[39] Swigris JJ, Wilson SR, Green KE et al. Development of the ATAQ-IPF: a tool to assess quality of life in IPF. Health Qual Life Outcomes. Jul 31 2010;8:77. 10.1186/1477-7525-8-77 20673370 10.1186/1477-7525-8-77 PMC2920246

[40] Khor YH, Palm A, Wong AW, et al. Effects of long-term oxygen therapy on acute exacerbation and hospital burden: the national DISCOVERY study. Thorax. 2025;80(6):378-384. 10.1136/thorax-2023-221063 10.1136/thorax-2023-221063 40113248

