# Supplementary material for: Real-world clinical and economic outcomes associated with supplemental oxygen therapy use among patients with fibrosing interstitial lung disease in the United States
Source: BMC Pulm Med. 2025 Nov 14;25:526. doi: 10.1186/s12890-025-03909-1 (PMC12619349; doi:10.1186/s12890-025-03909-1)

**Supplementary Tables and Figures**

**Supplementary Figure 1. Patient Identification and Attrition Diagram**

**
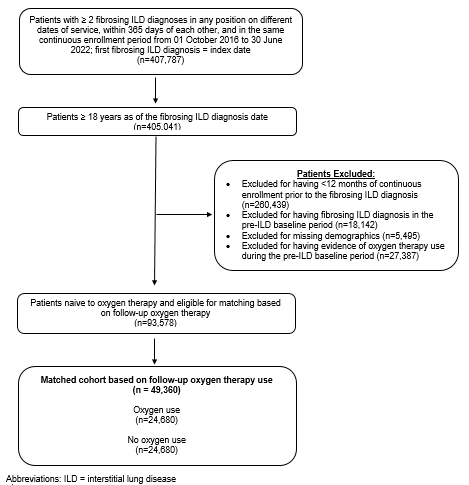
**

**Supplementary Figure 2. Time-to-probable acute exacerbation**


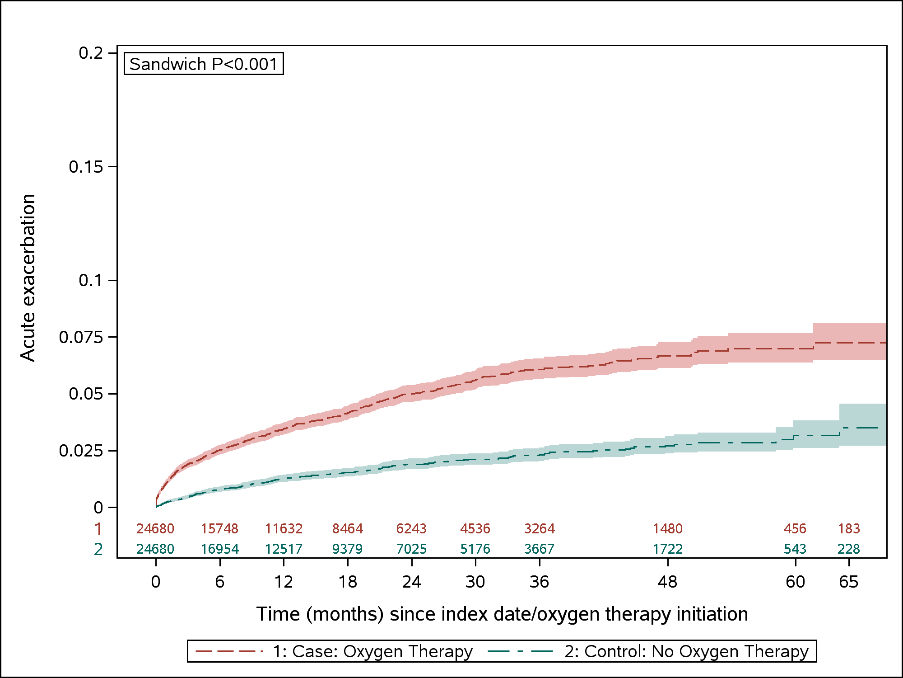


**Note:** Probable acute exacerbation is defined as the date of an imaging claim with a diagnosis for dyspnea or acute worsening, and no claims for an alternate cause, in the 30 days prior to (and including) imaging date; with at least 3 consecutive days with a steroid dosage of ≥60 mg/day between 15 days prior to, and up to 15 days after the imaging date. After the exacerbation date has been identified, the next date eligible for an exacerbation is an imaging date that occurs 16 days after the initial exacerbation; any additional imaging dates in the 15-day post-exacerbation were considered extensions of the initial exacerbation.

**Supplementary Figure 3. Time-to-all-cause inpatient visits**


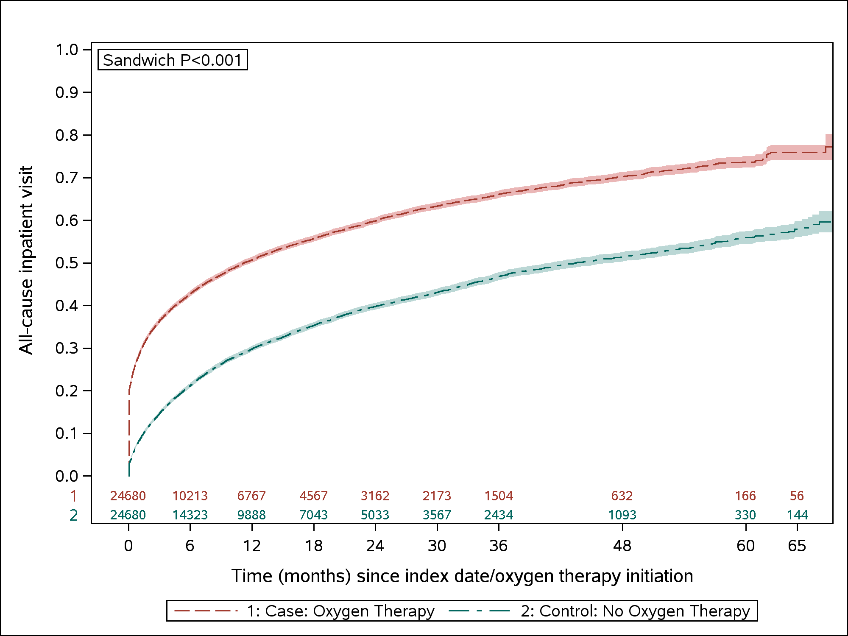

Supplement: Supplementary file 1 — Supplementary file1 (DOCX 150 KB) [file 12890_2025_3909_MOESM1_ESM.docx]
